# Patulin Detoxification by Recombinant Manganese Peroxidase from *Moniliophthora roreri* Expressed by *Pichia pastoris*

**DOI:** 10.3390/toxins14070440

**Published:** 2022-06-29

**Authors:** Shuai Wang, Xiaolu Wang, Leena Penttinen, Huiying Luo, Yuhong Zhang, Bo Liu, Bin Yao, Nina Hakulinen, Wei Zhang, Xiaoyun Su

**Affiliations:** 1 Biotechnology Research Institute, Chinese Academy of Agricultural Sciences, Beijing 100081, China; wangshuai9873@163.com (S.W.); zhangyuhong@caas.cn (Y.Z.); liubo01@caas.cn (B.L.); 2 State Key Laboratory of Animal Nutrition, Institute of Animal Sciences, Chinese Academy of Agricultural Sciences, Beijing 100193, China; wangxiaolu@caas.cn (X.W.); luohuiying@caas.cn (H.L.); binyao@caas.cn (B.Y.); 3 Department of Chemistry, Joensuu Campus, University of Eastern Finland, FIN-80101 Joensuu, Finland; leena.penttinen@uef.fi (L.P.); nina.hakulinen@uef.fi (N.H.)

**Keywords:** patulin, mycotoxin, manganese peroxidase, apple juice, detoxification

## Abstract

The fungal secondary metabolite patulin is a mycotoxin widespread in foods and beverages which poses a serious threat to human health. However, no enzyme was known to be able to degrade this mycotoxin. For the first time, we discovered that a manganese peroxidase (*Mr*MnP) from *Moniliophthora roreri* can efficiently degrade patulin. The *Mr*MnP gene was cloned into pPICZα(A) and then the recombinant plasmid was transformed into *Pichia pastoris* X-33. The recombinant strain produced extracellular manganese peroxidase with an activity of up to 3659.5 U/L. The manganese peroxidase *Mr*MnP was able to rapidly degrade patulin, with hydroascladiol appearing as a main degradation product. Five mg/L of pure patulin were completely degraded within 5 h. Moreover, up to 95% of the toxin was eliminated in a simulated patulin-contaminated apple juice after 24 h. Using *Escherichia coli* as a model, it was demonstrated that the deconstruction of patulin led to detoxification. Collectively, these traits make *Mr*MnP an intriguing candidate useful in enzymatic detoxification of patulin in foods and beverages.

## 1. Introduction

Patulin (PAT) is a secondary metabolite and a food-born mycotoxin produced by at least 60 different filamentous fungi including *Penicillium expansum*, *Penicillium shell*, *Penicillium clavum*, and *Aspergillus clavatus* [1]. This mycotoxin has been detected in many kinds of fruits (such as apples, pears, grapes, kiwifruit, blueberries, and peaches) and their products (such as juice, jam, and cider) [2,3,4]. Based on cellular and animal toxicological studies, it has been found that patulin can cause genotoxicity, embryonic toxicity, cytotoxicity, neurotoxicity, immunotoxicity, carcinogenicity, and teratogenicity [5]. At the molecular level, patulin induces DNA damage, leading to cell-cycle arrest, which inhibits the activity of cell survival proteins and induces apoptosis until cell death [5]. Because of its toxicity and high frequency of contamination, the World Health Organization (WHO), some European countries, the Food and Drug Administration of the United States (FDA), and the Ministry of Health of China have all set up their recommended maximum concentrations of patulin in foods and beverages. For example, 50 μg/kg, 25 μg/kg, and 10 μg/kg of patulin are allowed in apple juice, solid apple products, and fruit baby foods, respectively, in the European Union [6]. However, it was noted that, although legal provisions enforced in Serbia are in line with the EU regulations, from the 142 kinds of fruit juices (apple or multiple fruits) collected from the market in three consecutive years (2013–2015), patulin was detected in 51.4% of the fruit juices, and 0.7% of the samples exceeded the legal limit of 50 μg/kg [7]. In Spain, when analyzing PAT in 161 apple juice, 77 solid apple food, and 146 apple baby foods, PAT was discovered in 42% of the apple sauce samples, 32% in multiple fruit plates, and 25% in apple juice [8]. In Italy, 65% of 120 fruit plates and fruit paste samples tested positive for patulin [9]. The wide existence of patulin, in conjunction with its detrimental effects to the human health, points to the necessity to eliminate this mycotoxin in foods.

To remove the patulin contamination in foods, many trials have been carried out previously. These can be classified as physical, chemical, and biological treatments. Ultraviolet radiation is a physical means approved by both Canada and the United States for degradation of patulin in food [10], but the turbidity of apple juice and cider, plus the ascorbic acid present in large quantities, can significantly diminish the effect of this treatment. Treatment with chemicals such as ozone was reported to rapidly remove up to 98% of patulin within 1 min [11]. However, this manipulation can also generate new, undefined, chemicals and cause loss of important nutrients, thereby limiting its wide application in the food industry. These drawbacks prompt the scientific community to pursue other economically viable, efficient, safe, and environmentally friendly ways to remove the patulin and ensure food safety. Thus, biological decomposition of patulin in food by microorganisms or enzymes is emerging as an attractive alternative.

Up to now, the microorganisms that have been reported to be able to effectively degrade patulin include *Pichia caribbica* [12], the marine yeast *Kodameae ohmeri* [13], the biocontrol yeast *Rhodosporidium kratochvilovae* [14], and *Rhodotorula mucilaginosa* [15]. The rate of decomposition can reach as high as 97.66% [13]. Although the use of microbial cells has been proven to be an effective strategy, the presence of abundant residual cells and high concentrations of metabolites after treatment may change the final quality of the product. In essence, microbes transform patulin by their encoding enzymes. Unlike the microorganisms, the enzyme biocatalysts retain the ability to degrade patulin but do not introduce unwanted cells or their metabolites. Therefore, the use of detoxifying enzymes, which has the potential to ensure food safety and quality, should be a promising strategy in controlling patulin contamination in foods. However, even in the patulin-degrading microorganisms, the enzymes responsible for eliminating patulin have not been identified, thus impeding the use of an enzyme to detoxify patulin. Moreover, some microbes employ a strategy to import the mycotoxin and degrade it within the cell. This commonly involves the participation of expensive co-factors such as NAD^+^/NADH or NADP^+^/NADPH, which may not be economically viable in practical applications [16,17].

**Figure 1:**
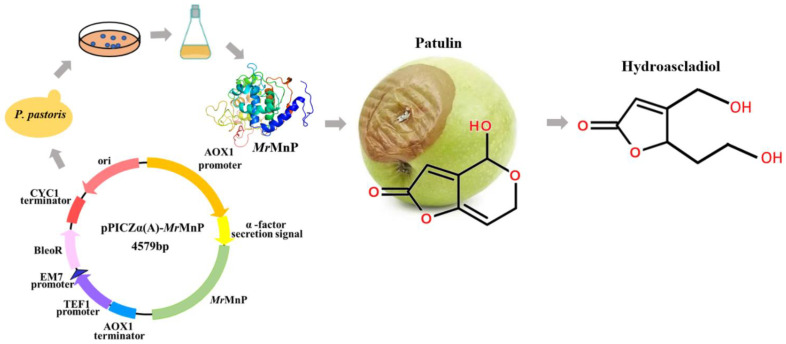
Schematic diagram showing the use of a recombinant *Mr*MnP expressed in *P. pastoris* to degrade and detoxify patulin, a mycotoxin commonly discovered in fruits.

Manganese peroxidases (MnP) are a kind of heme-containing peroxidases, which are generally produced by the lignocellulose-degrading basidiomycete filamentous fungi and symbolized by their ability to oxidize Mn^2+^ to Mn^3+^ [18]. In nature, the oxidized magnesium is chelated by dicarboxylic acids (such as oxalate [19]) excreted by the fungi, which can stabilize Mn^3+^ and penetrate into lignocellulose to attack the recalcitrant parts. Previously, we have demonstrated that manganese peroxidases can detoxify four major feed mycotoxins including aflatoxin, zearalenone, deoxynivalenol, and fumonisin [20], highlighting their potential to be used in feed. Moreover, this enzyme can act by either directly interacting with the substrate or indirectly through an oxidized Mn^3+^. Neither of the two ways involves the necessity of an expensive co-factor, saving the cost in mycotoxin degradation. However, the ability of MnP to degrade patulin remains unknown. Therefore, to understand whether a manganese peroxidase can degrade patulin and whether the degradation leads to detoxification, in this study, *Mr*MnP, a manganese peroxidase originating from *Moniliophthora roreri* [21] and recombinantly prepared in *Pichia pastoris*, was explored for its ability to degrade patulin (Figure 1). The nature of the degradation products was analyzed by mass spectrometry and the toxicity of the degradation products was determined.

## 2. Results and Discussion

### 2.1. Recombinant Production of the Manganese Peroxidase MrMnP

**Figure 2:**
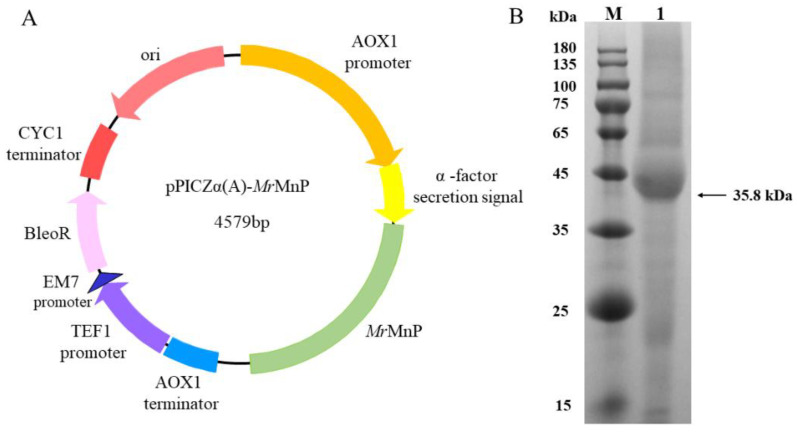
Expression of *Mr*MnP in *P. pastoris*. (**A**) The plasmid map of pPICZα(A)-*Mr*MnP. (**B**) SDS-PAGE analysis of the *Mr*MnP enzyme recombinantly produced in *P. pastoris*. The arrow indicates the recombinant *Mr*MnP protein. Lane M: protein molecular mass marker; 1: *Mr*MnP protein recombinantly produced in *P. pastoris*.

The most convenient way of characterizing the ability of a manganese peroxidase to degrade patulin would be to use a recombinant enzyme. However, the manganese peroxidases are notoriously difficult to be recombinantly produced, which might be, at least in part, ascribed to the presence of the heme prosthetic group in this kind of enzyme. Accordingly, many MnPs are expressed as insoluble, non-functional inclusion bodies in *Escherichia coli* and require tedious denaturation and refolding processes to obtain active enzymes [22,23,24]. However, in the commonly used eukaryotic microbial expression systems *P. pastoris* and *Aspergillus* spp., there were occasional reports indicating that a few MnPs could be functionally expressed, despite commonly being found in minor amounts [25,26,27,28,29,30]. The *M. roreri* MnP (*Mr*MnP) is such an enzyme that can be successfully produced in *P. pastoris* [21]. The gene encoding *Mr*MnP was thus artificially synthesized, cloned into the *Eco*RI and *Not*I restriction sites of pPICZα(A) to generate the recombinant plasmid pPICZα(A)-*Mr*MnP, and transformed into the *P. pastoris* X33 strain (Figure 2A). The transformant bearing the *Mr*MnP gene was first cultured in BMGY medium until the optical density of the culture at 600 nm (OD_600_) reached 6.0. Then, the medium was changed to BMMY to induce *Mr*MnP and the culture was continued for 5 d. The recombinant strain produced 271.4 and 3659.5 U/L of MnP (using ABTS as the substrate) in flask and fed-batch fermentations (Figure 2B), respectively. This is comparable to that described by Agathe et al. [21], thus providing a sound basis for subsequent enzymatic characterization and degradation of patulin.

### 2.2. The Di-Carboxylic Acids Play a Critical Role in Degradation of Patulin by MrMnP

**Figure 3:**
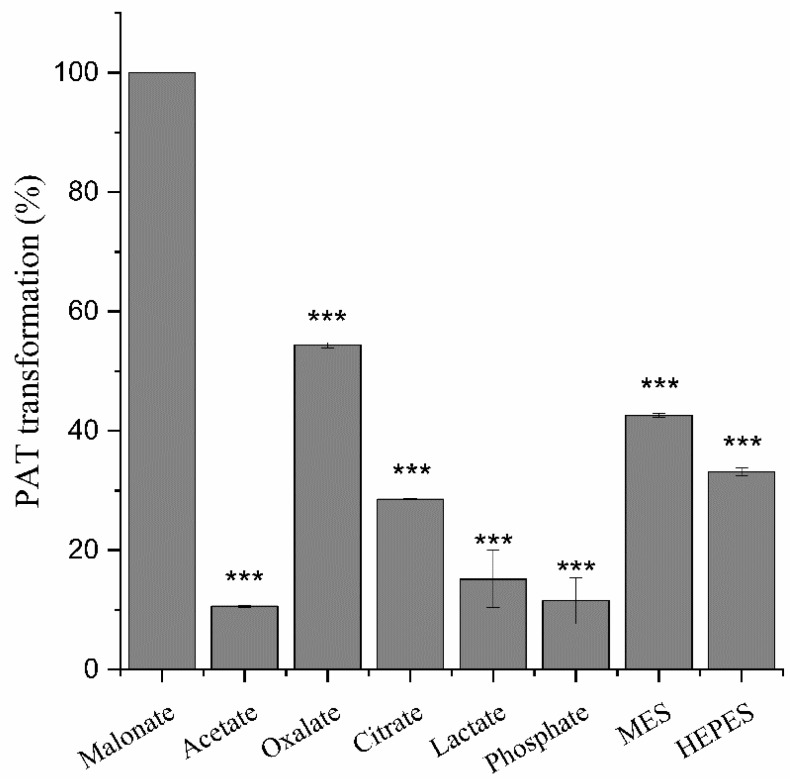
Effects of the buffer components on patulin transformation. The reactions were carried out by incubating 0.5 U/mL of *Mr*MnP with 5 mg/L of patulin in one of the buffers containing malonate, acetate, oxalate, citrate, lactate, phosphate, MES, and HEPES at 30 °C for 24 h. The data in the picture is the average ± standard deviation, *** *p* < 0.001, One Way anova test.

For an enzyme, the buffering system normally exerts a profound effect on its activity. Specifically, for manganese peroxidase, the buffer components may be directly involved in the reaction. For example, eight MnPs from different microbial sources have been demonstrated to be able to degrade four major feed mycotixins in presence of the di-carboxylic acid malonate [20], indicative of the potency of MnPs in detoxifying mycotoxins. However, in that study, the tested buffers were restricted to malonate, lactate, and acetate, while the effects of the buffers were only tested on aflatoxin B_1_ and zearalenone. Herein, the effects of the buffer components on degradation of patulin were systematically examined by including the tri-carboxylic acid (citrate), di-carboxylic acids (malonic acid and oxalic acid), the α-hydroxyl carboxylic acid (lactate), the mono-carboxylic acid (acetate), the inorganic acid (phosphate), and the zwitterionic buffers (MES, standing for 2-morpholinoethanesulphonic acid; and HEPES, standing for 2-[4-(2-hydroxyethyl)-1-piperazinyl] ethanesulfonic acid). In the previous study, it was noted that zearalenone, but not aflatoxin B1, could be transformed at a similarly high rate in the acetate buffer to that in the malonate buffer. Similarly, *Mr*MnP was highly efficient in degrading patulin, eliminating all the mycotoxin after 5 h of incubation in the malonate buffer (Figure 3). Contrary to the situation for zearalenone, patulin was only slightly degraded in the acetate buffer (10.6 ± 0.1%). The degradation in oxalate was not as high as that in malonate, but still obvious (54.3 ± 0.3%). The degradation was reduced to 28.5 ± 0.1% in citrate buffer and decreased to be marginal in the lactic acid (15.2 ± 3.4%) and phosphate (11.5 ± 2.7%). The reason for ineffective degradation may best be explained by the knowledge that Mn^2+^ cannot form stable chelates with either of the two acids. Degradation of mycotoxins in oxalate and citrate is of physiological relevance since, in nature, the fungi expressing MnPs can also produce these two compounds in their cellular metabolism [22]. Additionally, it has been reported that gluconic acid, cellobionic acid, and other organic acids as well, can aid manganese peroxidases to exert their degradation on lignin molecules [31]. This allows the fungi to use manganese peroxidases, in assistance with these naturally occurring molecules, to break the lignin barrier apart and capture energy from the plant cell wall polysaccharides [32]. Unexpectedly, there were 42.6 ± 0.3% and 33.1 ± 0.5% degradation in the MES and HEPES buffers (Figure 3), higher than those in acetate, citrate, lactate, and phosphate buffers. In the univariate analysis, there was an extremely significant difference (*p* < 0.001), indicating that the degradation of patulin was affected by the buffer.

### 2.3. Mn^2+^ Is Another Key Determinant in Degradation of Patulin by MrMnP

**Figure 4:**
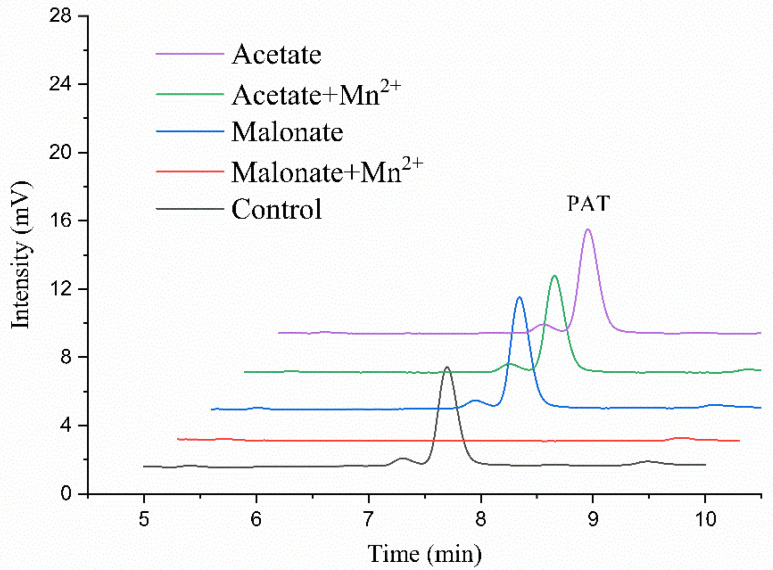
Mn^2+^ played an important role in degrading patulin. The reactions were carried out by incubating 0.5 U/mL of *Mr*MnP with 5 mg/L of patulin in the acetate or malonate buffer in absence or presence of Mn^2+^ at 30 °C for 24 h. Then the products were analyzed by HPLC.

Next, we sought to determine if the presence of Mn^2+^ would affect the activity of *Mn*MnP in patulin degradation. This is because the manganese peroxidases commonly have two substrate channels. One is the δ-heme edge, responsible for oxidation of hydrophobically-bound substrates including ABTS and phenolic compounds. The other one is the γ-heme edge, which is involved in catalysis of Mn^2+^ [33]. Patulin is a small chemical with ring structures, which might best fit for the first substrate channel, but could not enter the γ-heme edge. Incubation of *Mr*MnP with patulin in the acetate buffer, with or without Mn^2+^, did not lead to obvious degradation of patulin. The degradation rate is 1.1 ± 0.2% in acetic acid without Mn^2+^ and 10.6 ± 0.1% in the system containing Mn^2+^. The degradation was only observed in presence of both malonate and Mn^2+^ (Figure 4). The degradation rate reached 52 ± 0.3% at 2 h, and patulin was completely degraded at 5 h. These results collectively indicate that *Mr*MnP degraded patulin via a Mn^3+^-mediated indirect way, and patulin could not enter the δ-heme edge for degradation. The enzyme first catalyzed oxidation of Mn^2+^ to Mn^3+^, which then formed a chelate with malonate and subsequently oxidized patulin. Therefore, the enzyme’s activity against patulin appeared to be related to the stability of Mn^3+^ in complex with the buffer components in the reaction systems. The stability and reactivity of the Mn^3+^ ion are strongly dependent on the nature and concentrations of the Mn^3+^-complexing agents [18]. Both oxalate and malonate are dicarboxylic acids, but the degradation rate of patulin in malonate was higher, indicating that the chelate of Mn^3+^ and malonate is more stable and hence has higher activity for patulin. Based on this assumption, it was also suggested that, in the reactions with MES and HEPES serving the buffer systems, the newly generated Mn^3+^ was stabilized by these two buffers to a higher extent than that in acetate, citrate, lactate, and phosphate buffers. Although the reason for putatively increased Mn^3+^ stability remains unknown, it was noted that both chemicals have the sulfonate group, which could be involved in this stabilization.

### 2.4. MrMnP-Catalyzed Degradation of Patulin Led to Detoxification

**Figure 5:**
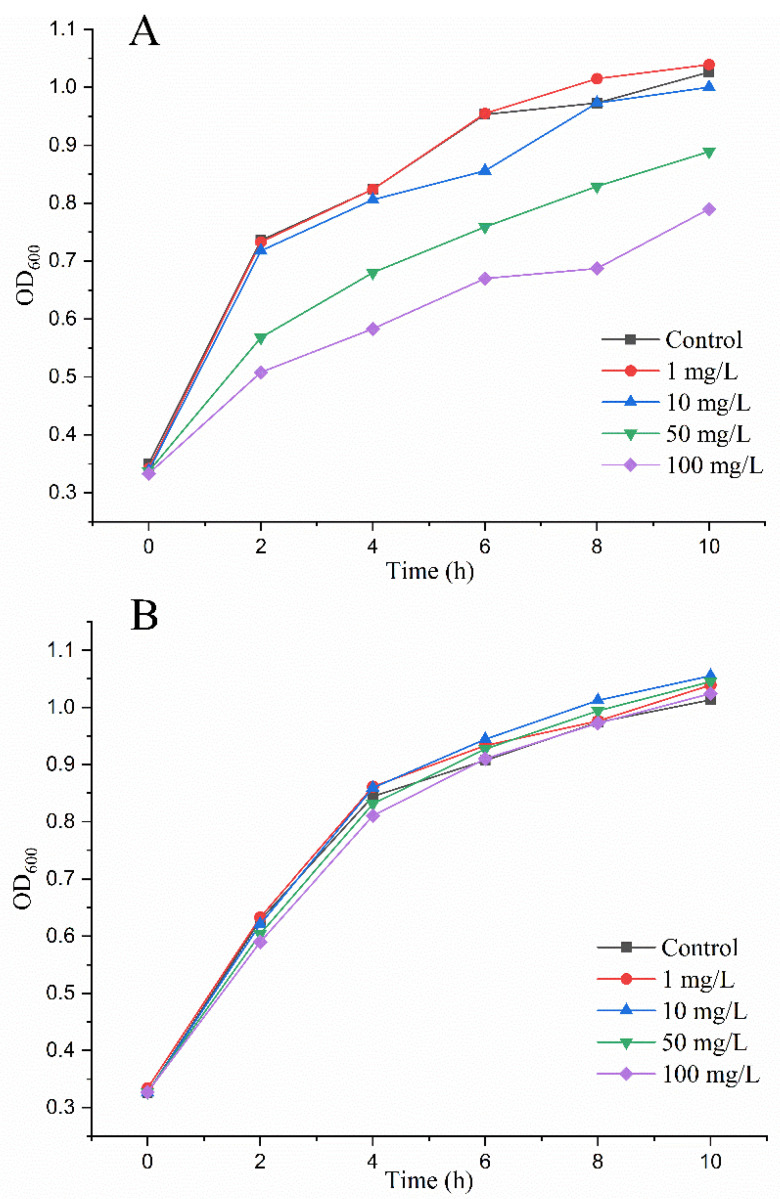
*Mr*MnP-catalyzed degradation of patulin led to detoxification. (**A**) Patulin was toxic to *E. coli* as demonstrated by retarded growth of the bacterium. A series of concentrations (1, 10, 50, and 100 mg/L) of patulin were added to equal amounts of *E. coli* and the culture was continued at 37 °C for 12 h. (**B**) *Mr*MnP-catalyzed degradation of patulin alleviated the retarding effect of patulin on *E. coli*. Patulin (1, 10, 50, and 100 mg/L) was first treated with 0.5 U/mL of *Mr*MnP at 30 °C for 24 h and then added to *E coli*.

The pre-requisite to apply *Mr*MnP in patulin detoxification is that the degradation products should have much less toxicity. *Escherichia coli* has been used as a microbial sensor system for successful monitoring of the toxicity of patulin [34]. In this study, increasing concentrations of untreated patulin were first incubated with *E. coli*. At 1 mg/L and 10 mg/L of patulin. There was no significant negative impact on the bacterial growth in a culturing period of 10 h. However, when 50 mg/L and 100 mg/L of patulin were added, the growth of *E. coli* was significantly retarded, as manifested by the drop of OD_600_ from 1.03 to 0.89 (for 50 mg/L) and 0.79 (for 100 mg/L) at 10 h after incubation (Figure 5A). These results indicated that *E. coli* could indeed be used to monitor the toxicity of patulin.

Patulin samples at final concentrations of 1, 10, 50, and 100 mg/L, respectively, were individually treated with 0.5 U/mL of *Mr*MnP. The HPLC analysis indicated that under all concentrations tested, the patulin was completely degraded (data not shown). It was observed that, at all these concentrations, the treated patulin no longer retarded the growth of *E. coli*, which suggested that the degradation of patulin by *Mr*MnP led to detoxification (Figure 5B).

### 2.5. Structural Analysis of the Degradation Products

**Figure 6:**
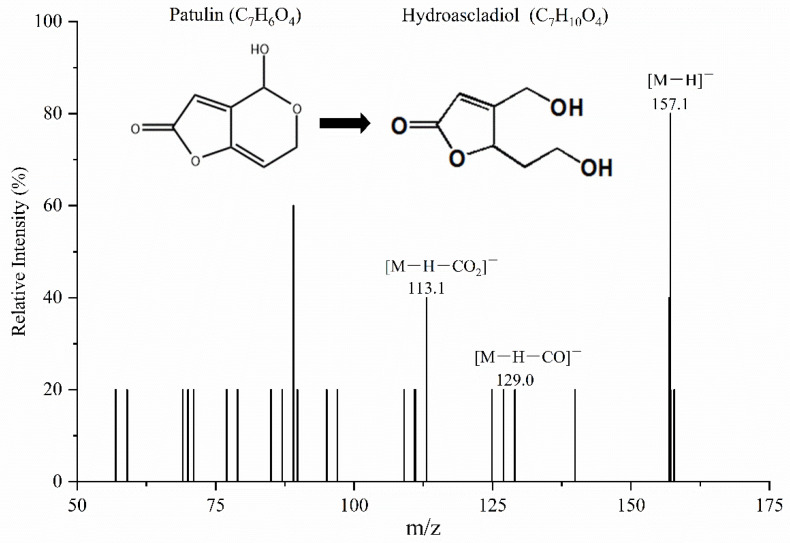
Identification of hydroascladiol as one major transformation product of patulin. PAT was incubated with 0.5 U/mL of *Mr*MnP in 50 mM malonate buffer (pH 5.0) supplemented with 1 mM MnSO_4_ and 0.1 mM H_2_O_2_ and the reaction was carried out at 30 °C for 8 h. The degradation products were analyzed by HPLC–MS/MS.

As *Mr*MnP-catalyzed degradation of patulin led to detoxification, it would be interesting to know the chemical nature of the degradation products. Degradation and detoxification of mycotoxins are a process that, in essence, involves the transformation of mycotoxins into less-toxic or even non-toxic compounds [35]. Therefore, the degradation products of *Mr*MnP on PAT were further identified by UPLC-MS/MS. It was found that hydroascladiol (5-(2-hydroxyethyl)-4-(hydroxymethyl)furan-2(5*H*)-one) was one of the main intermediate degradation products of PAT. The parent ion appeared at *m*/*z* 157.1 [M−H]^−^, producing daughter ions of 129.0 [M−H−CO]^−^ and 113.1 [M−H−CO_2_]^−^ (Figure 6). Daughter ions were produced by continuous loss of carbon dioxide [36]. In a previous study using *Lactobacillus plantarum* to degrade PAT, hydroascladiol was also obtained as the degradation product [37]. However, other intermediates including (E)-ascladiol and (Z)-ascladiol identified in their study were not discovered in our study. The toxicity of patulin is related to the hemiacetal and lactone rings in its structure. The generation of hydroascladiol, and hence concurrent destruction of the hemiacetal ring, can significantly reduce the toxicity of patulin [38]. With the prolonged reaction, hydroascladiol was further diminished (data are not shown), leading to further destruction of the lactone acid in hydroascladiol and lower toxicity. This is consistent with the observed decreased toxicity of the degradation products on *E. coli*.

### 2.6. Degradation in a Simulated Patulin-Contaminated Apple Juice

**Figure 7:**
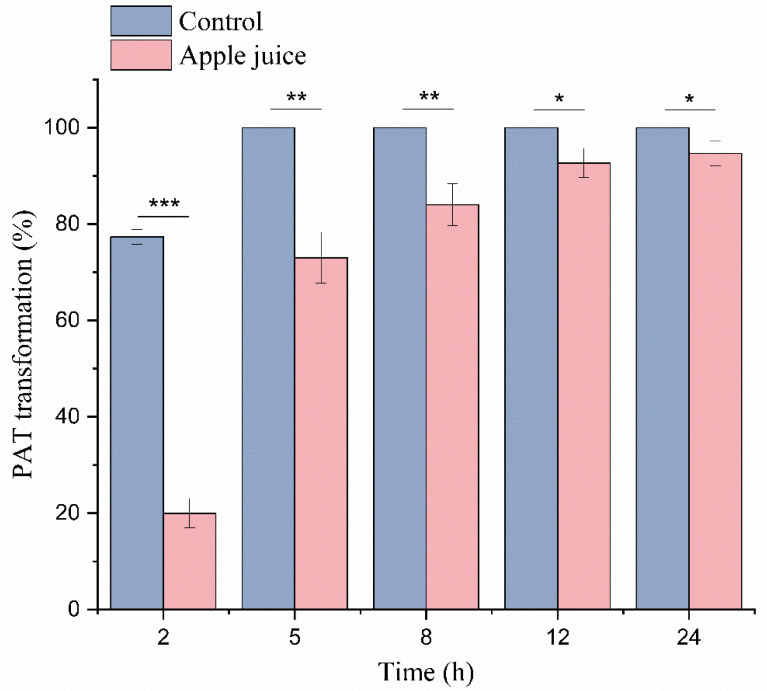
*Mr*MnP efficiently catalyzed degradation of patulin in a simulated patulin-contaminated apple juice. The reactions were carried out by incubating 0.5 U/mL of *Mr*MnP with 5 mg/L in absence (or presence) of apple juice. The samples were periodically taken out for HPLC analysis. The data in the picture is the average ± standard deviation, *** *p* < 0.001, ** *p* < 0.01, * *p* < 0.05, One Way anova test.

The ability of an enzyme to degrade a mycotoxin does not necessarily mean that the enzyme can efficiently degrade the mycotoxin in real foods/feeds. This is because the foods or feeds contain numerous components that could either adsorb the mycotoxins or act as competitors or inhibitors of the enzyme. For example, lignin phenolic compounds are naturally the substrate of MnPs [39,40,41,42]. In addition, many mycotoxins including aflatoxin B1, ochratoxin A, and zearalenone all have high affinity for lignocellulose, which also hinders the degradation process [43]. Therefore, to investigate whether *Mr*MnP can degrade patulin in the real environment, i.e., in foods with possibly interfering components, patulin was added to apple juice which then acted as a simulated patulin-contaminated beverage. In the control group, when no apple juice was added, rapid degradation of patulin was observed: the degradation rate was 76.8 ± 1.2% at 2 h of incubation and reached 100% after 5 h (Figure 7). When apple juice was present, the degradation rate of patulin was decreased to 19.8 ± 2.5% after incubation for 2 h. However, the degradation rate increased to 72.1 ± 4.3% after 5 h of incubation and then steadily increased to 95 ± 2.1% after 24 h. In the univariate analysis of 24 h data, there was a significant difference (*p* < 0.05). Therefore, it was evident that some components negatively affected degradation of patulin. However, the degradation rate of patulin was still highly comparable to that in absence of apple juice at 12 h and 24 h of incubation. Therefore, *Mr*MnP-catalyzed degradation of patulin can serve as an effective means to control the pollution of patulin in fruit juice.

Manganese peroxidases are present in many different microorganisms, such as *Irpex lacteus* [44], *Rhizoctonia* sp. [45], *Stereum Ostrea* [46], and *Phanerochaete chrysosporium* [47]. Regardless of the source of manganese peroxidases, they can all catalyze the oxidation of Mn^2+^ to Mn^3+^, which can be stabilized by forming complexes with a specific chelator in the reaction system. Therefore, it is expected that manganese peroxidase from other sources can also degrade patulin, and these enzymes will be a rich resource of candidate MnPs with physiochemical properties satisfying the demands of practical applications.

## 3. Conclusions

*Mr*MnP can degrade patulin most rapidly in the malonate/Mn^2+^ system, with 0.5 U/mL enzyme completely removing 5 mg/L of pure patulin within 5 h. One major degradation intermediate was identified by mass spectrometry to be hydroascladiol. In a simulated patulin-contaminated apple juice, 95% of patulin was eliminated after a 24 h treatment. Use of the enzyme to eliminate patulin contamination in juice appears to be advantageous to other detoxifying strategies such as microbial cells. Importantly, as the degradation led to detoxification, *Mr*MnP, and perhaps other manganese peroxidases, may serve as candidates for enzymatic detoxification of patulin in foods and beverages. As MnPs are commonly fragile, and the expression level of *Mr*MnP is still not comparable to those of other proteins such as glycoside hydrolases, in future efforts should be made to improve the stability and expression level of MnPs to reduce the cost in juice detoxification.

## 4. Materials and Methods

### 4.1. Strains and Plasmids

The *Escherichia coli* Trans1-T1 (TransGen, Beijing, China) was used for gene cloning and plasmid propagation. The *E. coli* DH5α (Vazyme, Nanjing, China) was used to detect the residual toxicity of patulin after *Mr*MnP treatment. The yeast used for recombinant *Mr*MnP expression was the *Pichia pastoris* X-33 strain (Invitrogen, Carlsbad, CA, USA). The plasmid used for construction of the expression plasmid was pPICZα(A) (Invitrogen, Carlsbad, CA, USA).

### 4.2. Cloning and Expression of MrMnP

The coding sequence of the *Mr*MnP manganese peroxidase gene (GenBank accession number: ESK95360.1) from *M. roreri* was codon-optimized according to the codon bias of *Pichia pastoris* and synthesized by the GenScript Biotech Corp. (Nanjing, China). Then, the *Mr*MnP gene was amplified from the synthesized gene by gene specific primers MrMnP-F and MrMnP-R (MrMnP-F: 5′-GCGGAATTCGCTGTTCCACAAAGAGTTGCTT-3′, where the underlined sequence indicates the *Eco*RI restriction site; MrMnP-R: 5′-GCGGCGGCCGCAGATGGTGGAACAGCTGGAAC-3′, where the underlined sequence indicates the *Not*I restriction site). The PCR product was treated with *Eco*RI and *Not*I and ligated into the expression vector pPICZα(A) pre-digested with the same two restriction enzymes to generate the recombinant plasmid pPICZα(A)-MrMnP, which was transformed into *E. coli* Trans1-T1 for cloning and sequencing. The integrity of the recombinant plasmid was confirmed by DNA sequencing. Then, the *Dra*I-linearized plasmid was transformed into the *P. pastoris* X33 competent cells by electroporation. Clones were selected on YPDS agar-plates including 100 µg/mL of zeocin.

A single colony of the transformant bearing the *Mr*MnP gene was inoculated into 10 mL of the YPD (yeast potato dextrose) medium, cultured at 30 °C overnight with an agitation of 200 rpm, and then transferred to 50 mL BMGY medium (containing 1% yeast extract, 2% peptone, 1% glycerol, 1.34% YNB, 100 mM sodium phosphate buffer pH 6, 0.4 mg/L biotin). The culture was continued at 30 °C and 200 rpm until the optical density of the culture at 600 nm (OD_600_) reached 6.0. The cells were then collected by centrifugation and suspended in 50 mL BMMY medium (containing 1% yeast extract, 2% peptone, 1% methanol, 1.34% YNB, 100 mM sodium phosphate buffer pH 6.0, 0.4 mg/L biotin). Methanol was added every 24 h to a final concentration of 1% (*v*/*w*) and the induction of enzyme expression was continued at 30 °C for 5 d. The secreted crude *Mr*MnP enzyme was collected from the culture supernatant.

### 4.3. Fed-Batch Fermentation of MrMnP in a Bioreactor

The X33 transformant integrated with the *Mr*MnP gene was inoculated in 50 mL YPD and incubated at 30 °C for 48 h with shaking at 220 rpm. This “primary seed” culture was transferred into another three 200 mL fresh YPD medium (with a ratio of one tenth, *v*/*v*) and the culture was continued overnight, which served as the “secondary seed”. This secondary seed culture was added into a bioreactor filled with 6 L of minimal salt medium, and the culture parameters were set with the pH to 4.0, temperature to 30 °C, and the rotation speed to 300 rpm. After the carbon source in the culture medium was exhausted, a mixture of glucose/methanol (glucose: 40%; glucose:methanol = 6:1) and 75 μM heme solution were added at a flow rate of 36 mL/(L h). The fermentation lasted for 120 h, during which the heme solution was replenished appropriately.

### 4.4. Degradation of Patulin by MrMnP

To determine the ability of *Mr*MnP to degrade patulin, the manganese peroxidase activity was first calibrated using 2,2′-azino-bis (3-ethylbenzothiazoline-6-sulfonic acid) (ABTS), which was carried out by monitoring the oxidation of ABTS (ε420 = 36,000 M^−1^ cm^−1^) at 420 nm in a buffer containing 50 mM malonate, 1 mM ABTS, 1 mM MnSO_4_, and 0.1 mM H_2_O_2_ (pH 5.0 and 25 °C) as described in Qin et al. [48]. Then, *Mr*MnP with an activity of 0.5 U/mL against ABTS was incubated with 5 mg/L of patulin in 50 mM malonate buffer, 1 mM MnSO_4_, and 0.1 mM H_2_O_2_. The reaction was carried out at 30 °C. At the end of the reaction, 3 volumes of methanol were added to the mixture for termination and the reaction products were analyzed by HPLC.

### 4.5. Effect of the Buffer Componesnts on Degradation of Patulin by MrMnP

To determine the effect of the buffer components on the degradation of patulin, the malonate (50 mM) was replaced with one of the other buffers, which included acetate, lactate, citrate, oxalate, phosphate, MES, and HEPES. The pH of the buffer was adjusted to 5.0 in all tested reaction systems. The reaction was carried out at 30 °C for 24 h. The reaction products were analyzed by HPLC and the degradation rate of patulin was then determined.

### 4.6. Effect of Mn^2+^ on Degradation of Patulin

In order to study the effect of Mn^2+^ on the degradation of patulin, the degradation rate of patulin in the malonate and acetate systems with or without Mn^2+^ was tested. All the reactions were carried out at 30 °C, and the samples were periodically (2 h, 5 h, 8 h, and 24 h) taken out for analysis of the degradation rate of patulin.

### 4.7. Toxicity Assay

The *E. coli* DH5α was used as a microbial sensor system to determine if the *Mr*MnP-catalyzed degradation products of patulin would still have toxicity [34]. *E. coli* was cultured in a Luria–Bertani broth (LB: 1.0% tryptone, 0.5% yeast extract, and 1.0% sodium chloride) to an OD_600_ of 0.3. Then, 0.5 U/mL of *Mr*MnP was added to varying concentrations (0, 1, 10, 50, and 100 mg/L) of patulin and the reaction was incubated at 30 °C for 24 h. Then, 5 mL of *E. coli* cells were mixed with 1 mL of patulin treated with or without *Mr*MnP. The growth of *E. coli* was carried out at 37 °C in a 96-well microplate. At 0, 2, 4, 6, 8, and 10 h, the OD_600_ was measured as an indicator of the growth of *E. coli* and toxicity of patulin to the bacterium.

### 4.8. HPLC and LC-MS/MS Analyses

Degradation of patulin was analyzed by High-Performance Liquid Chromatography (HPLC), which was carried out using a SHIMADZU 20A series instrument (Kyoto, Japan) with an Agilent ZORBAX SB-C18 column (5 µm, 4.6 mm × 250 mm) (Santa Clara, CA, USA). The elution condition included the use of 10% acetonitrile as the mobile phase, and the flow rate was set to 0.75 mL/min. Patulin was monitored under ultraviolet light of wavelength 276 nm.

The degradation products of patulin were further analyzed by using LC-MS/MS, which was carried out by coupling a SHIMADZU Nexera UHPLC system (Kyoto, Japan) to an AB-SCIEX 5600+ Triple TOF mass spectrometer (Waltham, MA, USA). The chromatographic column was XBrige BHE C18 (2.5 μm, 2.1 mm × 150 mm) and the column temperature was 40 °C. The mobile phase A was acetonitrile and the mobile phase B was 0.1% formic acid. One μL of the sample was injected and the flow rate was 0.3 mL/min. The elution procedure was as follows: initial 10% phase A; 0.5 min, 10% phase A; 1.5 min, 50% phase A; 5.0 min, 90% phase A; 6.0 min, 90% phase A; 6.2 min, 10% phase A; 8.0 min, 10% phase A. The detection conditions of mass spectrometry were as follows: negative ion; TOF-mass (Da) 50–500; ion source: Duo Spray Ion Source; ion source gas 1:50; ion source gas 2:50; curtain gas: 25; temperature: 450, IonSpray voltage floating (ISVF): −4500; declustering potential: −57.0; collision energy: −12.0; accumulation time: 0.1 s. ion scanning conditions: declustering potential: −80.0; collision energy: −30.0; collision energy spread: 0; ion release delay: 67; ion release width: 25.

### 4.9. MrMnP-Catalyzed Degradation of Patulin in a Simulated Patulin-Contaminated Apple Juice

The commercially available fresh apple juice (Huiyuan, Beijing, China) was obtained from a local retail market. No patulin was detected in this juice by HPLC analysis. Patulin was added to this product to a final concentration of 5 mg/L to simulate mycotoxin-contaminated juice. Then, the reaction was carried out by adding a final concentration of 0.5 U/mL of *Mr*MnP to the juice in the presence of 50 mM of malonic acid solution and 1 mM of MnSO_4_. The reaction was initiated by addition of 0.1 mM of H_2_O_2_. At 0, 2, 5, 8, and 24 h, samples were taken out for HPLC analysis.

### 4.10. Statistical Analysis

The results of three repetitions were expressed as mean ± standard deviation (SD). The data were analyzed by one-way analysis of variance (ANOVA).

## Data Availability

The data presented in this study are available on request from the corresponding author.

## Abbreviations

PAT	Patulin
MnP	Manganese Peroxidases
ABTS	2,2′-Azino-bis (3-ethylbenzothiazoline-6-sulfonic acid)
H_2_O_2_	Hydrogen Peroxide
MES	2-Morpholinoethanesulphonic acid
HEPES	2-[4-(2-Hydroxyethyl)-1-piperazinyl] Ethanesulfonic acid
